# Neural Peptide *α*-CGRP Coregulated Angiogenesis and Osteogenesis via Promoting the Cross-Talk between Mesenchymal Stem Cells and Endothelial Cells

**DOI:** 10.1155/2022/1585840

**Published:** 2022-06-16

**Authors:** Zongxin Shi, Shikun Wang, Jiechao Deng, Zishun Gong

**Affiliations:** Department of Orthopedic Surgery, Beijing Fangshan District Liangxiang Hospital and Liangxiang Teaching Hospital of Capital Medical University, No. 45, Gongchen Ave., Liangxiang, Fangshan Dist. Beijing, Beijing 102488, China

## Abstract

**Background:**

The coupled vascularization and bone remodeling are key steps during bone healing, during which the cross-talk between mesenchymal stem cells (MSCs) and endothelial cells plays vital roles. Evidence indicates the well-characterized neuropeptide Calcitonin Gene-Related Peptide-*α* (CGRP) is proven to play an important role during bone regeneration. However, the regulatory effects of *α*CGRP on angiogenesis and osteogenesis, as well as underlying cellular and molecular mechanisms, remain unclear.

**Aim:**

The present study was performed to verify the availability of the CGRP for osteogenic capacity in MSCs and explore its potential underlying molecular mechanism. After that, the promoted angiogenic effect of CGRP as well as its underlying mechanisms was studied.

**Methods and Results:**

The results showed that CGRP could significantly increase the cyclic adenosine monophosphate (cAMP) level and promote the osteogenesis ability of MSCs via cAMP/PKA signaling pathway. Direct exposure to CGRP increased nitric oxide synthase expression, the release of NO, tube formation, and wound healing of human umbilical vein endothelial cells (HUVEC). The CGRP-treated MSCs were observed with high expression levels of angiogenic factors, such as bFGF and VEGF-*α*; the conditioned medium derived from CGRP-treated MSCs was also able to promote tube formation and transmembrane migration of HUVECs.

**Conclusion:**

These findings demonstrate the coregulated angiogenesis and osteogenesis effects of CGRP, especially for its regulation effects on the cross-talk between mesenchymal stem cells and endothelial cells.

## 1. Introduction

The regeneration of large bone fracture remains to be a serious clinical challenge for orthopedic surgeons as many people suffer from bone diseases caused by trauma, infection, arthritis, tumors, osteonecrosis, osteoporosis, and metabolic bone disease [1–3]. In the bone tissue, sensory nerves have been highlighted for their potential to heal damaged bone tissue. Some neuropeptides or neurogenic factors, such as nerve growth factor (NGF), tropomyosin receptor kinase A (TrkA), neurofilament 200 kDa (NF200), and calcitonin gene-related polypeptide-*α* (CGRP-*α* or CGRP), were proved to be critically involved in bone metabolism, osteogenesis, and bone healing [4, 5]. Among them, CGRP is suggested to be one of the most important osteoanabolic peptides [6].

CGRP, a 37-residue peptide produced in specific neurons by alternative splicing of the calcitonin gene, is an important neuropeptide involved in bone growth and metabolism, which is widely distributed throughout the central and peripheral nervous systems [7]. After being synthesized by *sensory* nerves, CGRP could be released from the terminal of these nerves in bone tissue following neuronal depolarization and exerts its biological functions during bone regeneration [8, 9]. Numerous effects of CGRP in bone-related cells have indicated its potential regulation effects on bone growth and metabolism. Recently, studies have provided evidence that CGRP may play a key role in promoting recruitment, stimulating the proliferation and differentiation of osteoblastic cells, and improving bone fracture healing and regeneration [10, 11]. CGRP is also proven to enhance osteogenic differentiation of periosteum-derived stem cells (PDSCs) by inducing activation of cAMP-responsive element-binding protein 1 (CREB1) and SP7 (also known as osterix) [12], which reveals the important roles of CGRP in promoting osteogenic differentiation and bone regeneration. Recently, some papers also verified the therapeutic potential of CGRP for bone regeneration in orthopedics [13, 14].

Similar to skeletal development, bone healing or regeneration evolves the coordination of multiple events including migration, differentiation, and activation of many cell types in the injury site [15, 16]. Despite many important roles of osteoblastic cells, the development of microvasculature and microcirculation is critical for regenerated functional new bone [17]. Apart from the transportation of oxygen, nutrients, soluble factors, and numerous cell types, blood vessels could also provide so-called angiocrine signals to control the bone healing process. Evidence also indicates that the coupled angiogenesis to osteogenesis is key steps during bone healing. Especially, the cross-talk between endothelial cells and osteogenic cells, such as mesenchymal stem cells (MSCs) and osteoprogenitors, plays vital roles [18, 19]. During new bone formation, self-renewing mesenchymal stem cells were ordered to differentiate into osteoprogenitors, osteoblasts, and eventually osteocytes under complex stimuli. Except for direct differentiation into functionally bone cells for fracture healing, MSCs were also observed with broader paracrine functions via secreted bioactive factors, especially for angiogenic factors. What is more, previously published papers also proved the vital promotion roles of CGRP on angiogenesis and osteogenesis in bone healing [13, 14, 20]. However, the underlying cellular and molecular mechanisms for the coregulation effects of CGRP on angiogenesis and osteogenesis remain unclear.

In this paper, the angiogenesis and osteogenesis effects of CGRP were investigated *in vitro*, and the underlying molecular mechanisms were studied as well, especially for the regulation effects of CGRP on cross-talk between mesenchymal stem cells and endothelial cells.

## 2. Materials and Methods

### 2.1. Materials

Human MSCs and HUVEC were purchased from Saiye Co. (China). CGRP was purchased from Abcam Co. (USA, cat. #ab47101). Cell counting kit-8 (CCK-8) kit, p-nitrophenyl phosphate, and bicinchoninic acid (BCA) assay kit were obtained from Beyotime Biotechnology Co. (Jiangsu, China). Alizarin red sodium salt was obtained from Alfa Aesar Co. (Tianjin, China). The CGRP receptor antagonist BIBN4096BS was purchased from Shanghai Haoyuan Chemexpress Co. (Shanghai, China). Phosphate buffer solution (PBS) was provided by Dingguo Biotechnology Co. (Beijing, China). Other chemicals were purchased from Oriental Chemical Co. (Chongqing, China).

### 2.2. Cell Culture

MSCs were cultured in tissue culture polystyrene (TCPS) dishes in alpha-MEM supplemented with 10% fetal bovine serum, penicillin (100 U/ml), and streptomycin (100 *μ*g/ml) and passaged by 0.05% trypsin digestion. MSCs at passage 3 were used in our study. HUVECs were cultured in tissue culture polystyrene (TCPS) dishes in Medium 199 (M199, GIBCO, USA) containing 15% fetal bovine serum (GIBCO, USA), 5 ng/ml recombinant human EGF (Sigma), 0.75 units/mL heparin sulfate (Sigma), 0.75 units/mL hydrocortisone hemisuccinate (Sigma), 50 *μ*g/mL of ascorbic acid, and passaged by 0.05% trypsin digestion. HUVECs at passages 3 to 5 were used in the experiments.

### 2.3. Cell Viability

To estimate MSC proliferation viability, CCK-8 assay was applied. MSCs (2 × 10^4^ cells/mL) were seeded into different groups. 10^−12^ to 10^−7^ M CGRP were used. After culture for 2 days, cell proliferation was investigated via the CCK-8 assay. Briefly, at the prescribed time points, all groups were gently rinsed with a PBS solution 3 times. Next, a mixture solution (220 *μ*L) of fresh medium and CCK-8 solution (*v*/*v* = 10 : 1) was added to each well. After incubation for 1 h, the optical density (OD) was detected with a spectrophotometric microplate reader (Bio-Rad 680, USA) at a wavelength of 450 nm.

### 2.4. Detection of cAMP Secreted by MSCs

The MSCs were incubated with different concentrations of CGRP for 10 min at 37°C, or without treatment. The levels of cAMP were assayed using a commercial cAMP assay kit (Nuclear Medicine Laboratory of Shanghai University of Traditional Chinese Medicine, Shanghai, China), according to the protocol from the manufacturer.

### 2.5. Alkaline Phosphatase (ALP) Activity

The MSCs (2 × 10^4^ cells/cm^2^) were firstly seeded onto 24-well plates. The control group was incubated with *α*-MEM based culture medium only as mentioned above. The experimental groups were treated *α*-MEM based culture medium plus 10^−9^ mol/l CGRP for the CGRP group, or 10^−9^ mol/l CGRP and its receptor antagonist BIBN4096BS (10^−9^ mol/l) for the CGRP-BIBN group. The ALP staining and activity assay of MSCs were performed after culture for 7 and 14 days. For ALP staining, MSCs were fixed with 4% paraformaldehyde for 30 min and stained with the BCIP/NBT alkaline phosphatase staining kit, respectively. Then, images were recorded using an Olympus MVX10 MacroView (Japan). For the ALP activity assay, at the prescribed time points, MSCs adhered on different groups were lysed by 1% Triton X-100 for 30 min. The ALP activity and total BCA were measured using the p-nitrophenyl phosphate and BCA assay kit, respectively. The optical density (OD) values of ALP and BCA were detected with a spectrophotometric microplate reader (Bio-Rad 680, USA) at wavelengths of 490 nm and 570 nm, respectively.

### 2.6. Mineralization Assay

The mineralization assay was performed according to a previous study. Specifically, after 14 days of culture, the mineralization of MSCs (initial density of 2 × 10^4^ cells/mL) was investigated via Alizarin Red S staining (ARS). Alizarin Red S is considered as the gold standard for assessing calcium deposits in the mineralized extracellular matrix (ECM) of differentiated stem cells. ARS works through a chelation process during which calcium forms an Alizarin Red S-calcium complex, and the stain is directly proportional to the degree of mineralization [21]. Briefly, MSCs, rinsed with PBS 3 times, were fixed with 4% paraformaldehyde and stained with 40 mM Alizarin Red S (pH 4.1), respectively. The staining images were recorded using an Olympus MVX10 MacroView (Japan). For the quantitative assay, the stained cells were detached with 10% acetic acid (*v*/*v*) solution for 30 min, and then, the mixture solution was transferred into a vial. After vortexing for 30 s and heating (85°C) for 10 min, the excess acid was neutralized with 10% ammonium hydroxide. Finally, the optical density (OD) values were detected with a spectrophotometric microplate reader (Bio-Rad 680, USA) at 405 nm.

### 2.7. Quantitative Real-Time Polymerase Chain Reaction (qRT-PCR) Assay

The mRNA expressions of osteogenesis-related genes in MSCs (2 × 10^4^ cells/mL) were investigated via qRT-PCR. The MSCs were cultured for 14 days. Bio-Rad CFX Manager system was applied to perform qRT-PCR. The total RNA was extracted using the Trizol reagent. Then, transcription of first-strand cDNA was performed with an RNA extract kit (Bioteck Co.) and PrimeScript™RT reagent kit (Takara Co.). Two-step cycling amplification conditions were employed as follows: 95°C for 30 s, followed by 39 cycles of 95°C for 5 s and 60°C for 30 s. The related primers are displayed in Table 1, and *β*-actin was selected as the reference gene in this study.

### 2.8. Western Blotting

For western blotting experiments, the MSCs were lysed with Laemmli sample buffer (Bio-Rad Laboratories, CA, USA). The total protein content of the lysates was measured using the Pierce BCA Protein Assay Kit (ThermoFisher Scientific-Life Technologies, Carlsbad, CA, USA), followed by further heating at 100°C for 10 min. The samples were loaded onto 12% sodium dodecyl sulfate-polyacrylamide gels (SDS-PAGE) with equal amounts of total protein, electrotransferred to polyvinylidene fluoride (PVDF) membranes (Bio-Rad Laboratories, Inc.), and blocked with 1X TBST with 5% *w*/*v* nonfat dry milk at room temperature for 1.5 h. The membranes were incubated with primary antibodies overnight at 4°C. Rabbit monoclonal anti-CALCRL (calcitonin receptor-like receptor, 1 : 1000), anti-RAMP-1 (receptor activity modifying protein 1, 1 : 1000), anti-PKA (cAMP-dependent protein kinase, 1 : 1000), anti-pPKA (phospho-PKA, 1 : 1000), anti-CREB (cAMP response element-binding protein, 1 : 1000), anti-pCREB (phospho-CREB, 1 : 1000), and anti-GADPH (glyceraldehyde 3-phosphate dehydrogenase, 1 : 1000) primary antibodies from Cell Signaling Technology (CST, USA) were used. The blots were then exposed to the horseradish peroxidase-labeled secondary antirabbit antibody (1 : 5,000) for 2 h at room temperature. The proteins were visualized using an enhanced chemiluminescence kit (Thermo Fisher Scientific, MA, USA), and the band signals were detected using a gel imaging system (Syngene, Frederick, MD, USA). The intensities were quantified using Image J software, and the relative expression levels of certain proteins were calculated via band intensity normalization.

### 2.9. HUVEC F-Actin Staining

The HUVECs (2 × 10^4^ cells/cm^2^) were firstly seeded onto 24-well plates and incubated with M199 medium only (TCPS group), M199 medium containing 10^−9^ mol/l CGRP (CGRP group), or M199 medium containing 10^−9^ mol/l CGRP and its receptor antagonist BIBN4096BS (10^−9^ mol/l) (CGRP-BIBN group). After culture for 24 hours, the HUVECs were fixed with 4% paraformaldehyde for 15 min for F-actin staining. After being permeabilized for 15 min with 0.1% Triton X-100, the samples were incubated with 1.65 *μ*mol/l rhodamine-labeled phalloidin overnight at 4°C and washed three times with PBS. Nuclei were stained with 1 *μ*g/ml DAPI (PBS) for 15 min and washed three times with PBS. The cells were observed and analyzed by confocal microscopy.

### 2.10. Inducible Nitric Oxygen Synthase (iNOS) Staining of HUVECs

To evaluate the iNOS of HUVECs, the cells were fixed with 4% paraformaldehyde for 15 min after culture for 24 hours. After being washed with PBS, HUVECs were permeabilized for 15 min with 0.1% Triton X-100 and then blocked with goat serum for 1 hour. Cells were incubated with iNOS primary antibody (Catalog no. EPR1663, Abcam, Cambridge, UK) overnight at 4°C. After three washes in PBS, FITC marked secondary antibody (Catalog no. BA1105, Boster, Wuhan, China) was added to cells for further 1-hour incubation. The samples were washed three times with PBS, and nuclei were stained with 1 *μ*g/ml DAPI (PBS) for 15 min. The cells were observed and analyzed by confocal microscopy.

### 2.11. Nitric Oxide Release by Endothelial Cells

Nitric oxide (NO) release of HUVECs was measured by detecting the photometric means of its stable breakdown products. Shortly, Griess method was applied to detect NO concentration in culture medium using a Nitric Oxide Assay Kit (Beyotime Biotechnology, China). After culture for 24 hours, 50 *μ*L of the culture medium was diluted with 50 *μ*L of Griess reagent I and II. Nitrite concentration was measured at 540 nm and calculated accordingly.

### 2.12. Tube Formation Assays

HUVECs (5.0 × 10^4^) were seeded on growth factor-reduced Matrigel Matrix (Corning, USA) coated wells in a 96-well plate with or without CGRP and BIBN4096BS for 24 hours. Tube formation was observed with an inverted microscope, and images of random separate fields from each group were recorded. The tube numbers of “tube” were quantified.

### 2.13. Wound Healing Assay

To investigate the *in vitro* migration of HUVECs, a scratch wound assay model was applied. In short, HUVECs were seeded in 24-well plates at a density of 1.0 × 10^5^/well. After reaching 90% confluency, the cell layers were then scratched longitudinally with a P10 pipette tip to create a straight wound. The samples were washed with M199 for two times, and the remained cells were further cultured in M199 medium supplemented with 1% FBS for 12 hours. The wound area of HUVECs was observed with an inverted microscope at 0 and 12 hours, and images of random separate fields from each group were recorded. The wound area was calculated by manually tracing the cell-free area in captured images using Image-Pro Plus software (Media Cybernetics). The relative wound closure was expressed as the percentage of wound area change over time [22, 23].

### 2.14. Angiogenic Regulation Effects of MSCs

#### 2.14.1. Angiogenic Gene Expression Levels of MSCs

A total of 4 × 10^4^ MSCs per well were firstly seeded onto 24-well plates and incubated with *α*-MEM medium only (control group), *α*-MEM containing 10^−9^ mol/l CGRP(CGRP group), or *α*-MEM containing 10^−9^ mol/l CGRP and its receptor antagonist BIBN4096BS (10^−9^ mol/l) (CGRP-BIBN group), respectively, for 3 days. After that, qRT-PCR was performed to detect mRNA expression of angiogenic factors including bFGF, EGF, HIF-1a, and VEGF-a with the same method mentioned above.

#### 2.14.2. Paracrine Effects of MSCs on Endothelial Tube Formation

To investigate the paracrine effects of MSCs on tube formation of HUVECs, MSC-derived conditioned medium (CM) was firstly obtained from MSC cultures. A total of 1.6 × 10^5^ MSCs per well were firstly seeded onto 24-well plates and incubated for 3 days. After being washed 3 times with phosphate-buffered saline (PBS), the medium was replaced with serum-free M199 for 24 hours. The CM was concentrated and used as the HUVEC culture medium. HUVEC tube formation assay was performed as mentioned above.

#### 2.14.3. Transwell Experiment for HUVEC Migration Assay

To detect the paracrine effects of MSCs on the migration ability of HUVECs, a transwell coculture system was applied. In short, a total of 4 × 10^4^ MSCs per well were firstly seeded onto 24-well plates and incubated with *α*-MEM medium only (control group), *α*-MEM containing 10^−9^ mol/l CGRP (CGRP group), or *α*-MEM containing 10^−9^ mol/l CGRP and its receptor antagonist BIBN4096BS (10^−9^ mol/l) (CGRP-BIBN group), respectively, for 3 days. After being washed 3 times with PBS, the medium was replaced with 1% FBS + M199, and HUVECs were seeded at Matrigel Matrix pretreated upper chamber of the transwell with 8 *μ*m pores (Corning, USA). After being cocultured for 24 hours, the transmembrane migrated HUVECs were fixed with 4% paraformaldehyde and then stained with 1% crystal violet solution. The migrated cells were observed with an inverted microscope and calculated from random separate fields in each group.

### 2.15. Statistical Analysis

All data were present as means ± standard deviation (SD). The statistical analysis was carried out with OriginPro (8.0) via the one-way ANOVA followed by Dunnett's posttest for multiple comparisons. The confidence levels were set as 95% and 99%.

## 3. Results

### 3.1. Evaluation of the Cell Viability and cAMP Content for MSCs Treated with Different Concentrations of CGRP

**Figure 1:**
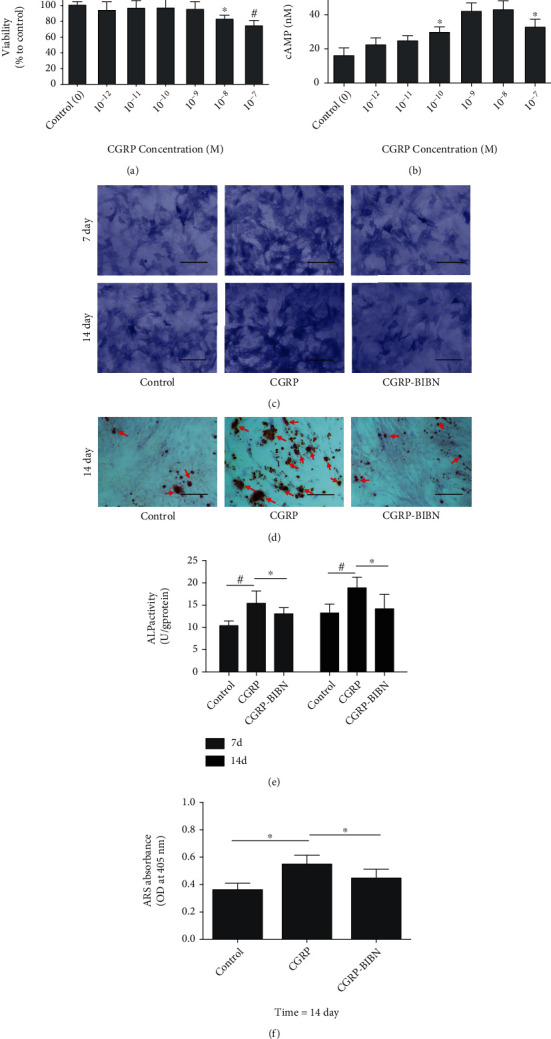
(a) Cell viability assay by CCK-8 assay for MSC proliferation in different concentrations of CGRP for 2 days (*n* = 6). (b) cAMP levels were released by MSCs cultured in different concentrations of CGRP for 2 days. (c) ALP staining of MSCs in different groups after culture for 4 and 7 days, respectively (scale bar: 250 *μ*m) and (e) ALP activity of MSCs cultured in different groups after culture for 4 and 7 days (*n* = 6). (d) Representative ARS stained the mineral deposition (red, red arrows) of MSCs after culture for 14 days (scale bar: 250 *μ*m). (f) Quantification of MSCs mineralization (*n* = 6) from different groups after culture for 14 days. ^∗^*p* < 0.05, ^#^*p* < 0.01.

To verify the regulation role of CGRP in osteogenesis of MSCs, the cell viability of MSCs treated with different concentrations of CGRP (10^−12^–10^−7^ M) was firstly determined by CCK-8 method. MSCs treated without CGRP were used as the control group. It could be found that the cell proliferation of MSCs cultured with 10^−12^–10^−9^ M CGRP was not affected during the first 2 days. However, MSCs grown in the 10^−8^ and 10^−7^ M CGRP groups displayed significantly lower (*p* < 0.05 or *p* < 0.01) cell viability than those treated without CGRP (Figure 1(a)). Therefore, a high concentration of CGRP might negatively affect the MSCs proliferation, and 10^−9^ M is the critical value of CGRP to inhibit cell viability of MSCs. To further investigate the cAMP content released by MSCs treated with different concentrations of CGRP (10^−12^–10^−7^ M), the levels of cAMP were measured by using a commercial cAMP assay kit. As shown in Figure 1(b), compared with the control group, the cAMP level was significantly promoted when MSCs were treated with high concentration of CGRP (10^−10^–10^−7^ M) (*p* < 0.05 or *p* < 0.01). However, the cAMP secretion treated with 10^−7^ M CGRP was inhibited compared with those treated with 10^−9^ and 10^−8^ M. Therefore, the most appropriate concentration of CGRP with 10^−9^ M was applied for further study.

### 3.2. CGRP Increases the Alkaline Phosphatase (ALP) Activity of MSCs

Alkaline phosphatase (ALP) catalyzes the hydrolysis of phosphate esters in the extracellular space and results in production of an organic radical and inorganic phosphate, which are prerequisites for osteoblast differentiation. Thus, ALP is normally considered to be an initial marker of osteoblast differentiation [24]. In order to investigate the regulation effect of the CGRP on early-stage osteogenic differentiation of MSC *in vitro*, ALP activity of MSCs from each group was evaluated. As shown in Figure 1(c), high ALP-positive intensity was observed in the CGRP group as compared to the other two groups at 7 and 14 days. Quantitative ALP analysis (Figure 1(e)**)** confirmed that CGRP could significantly increase the ALP activity of MSCs after culture for 7 and 14 days (*p* < 0.05 or *p* < 0.01). However, the MSCs pretreated with BIBN4096BS, a CGRP-receptor antagonist, were observed with significantly lower ALP activity (*p* < 0.05) in CGRP-BIBN group than in the CGRP-treated group (*p* < 0.05 or *p* < 0.01). These results indicated that CGRP could promote the early-stage differentiation of MSCs, and it could be effectively inhibited via CGRP-receptor antagonist pretreatment.

### 3.3. The Analysis of ECM Mineralization of MSCs Treated with CGRP

To investigate the late-stage osteogenic differentiation *in vitro*, ECM mineralization was investigated by Alizarin red staining after being cultured in different groups for 14 days. As shown in Figure 1(d), optical images of the ARS staining showed that more calcium deposits (red dots and red arrows) were observed in the CGRP group than those in the CGRP-BIBN group and the control group. Quantitative analysis (Figure 1(f)) results confirmed that MSCs in the CGRP group displayed the highest mineralization among all groups (*p* < 0.05). Therefore, the late-stage differentiation of MSCs was promoted in the presence of 10^−9^ M CGRP, and the CGRP-receptor antagonist pretreatment could inhibit the differentiation promotion effect of CGRP.

### 3.4. CGRP Promotes Osteogenic Gene Expression

**Figure 2:**
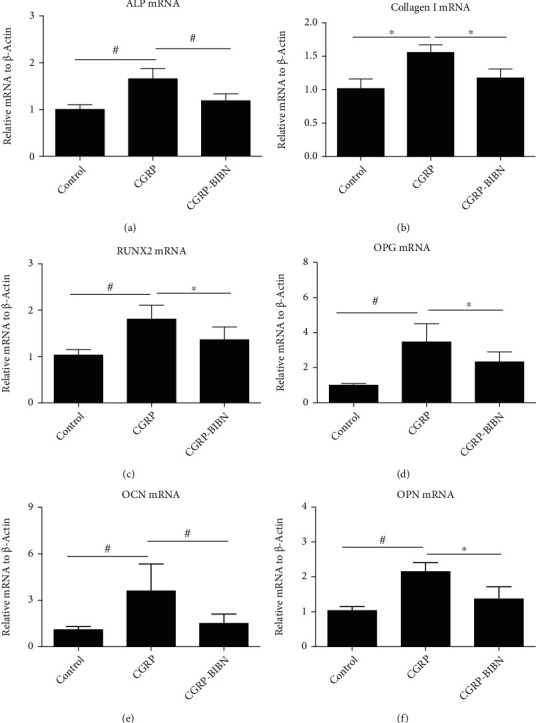
Relative osteogenic-specific mRNA expressions of MSCs cultured in different groups for 14 days. The value was normalized to *β*-actin (*n* = 6). ^∗^*p* < 0.05, ^#^*p* < 0.01.

To further confirm the enhanced osteoblast function at molecular level, gene expression analysis was performed using quantitative real-time polymerase chain reaction (qRT-PCR). The mRNA expressions of six bone formation related genes, including Runt-related transcription factor 2 (Runx2), collagen type I (Col I), osteopontin (OPN), osteocalcin (OCN), ALP, and osteoprotegerin (OPG) expressed by MSCs cultured in various groups, were measured after culture for 14 days (Figure 2). The results showed that MSCs in the CGRP group displayed significantly higher (*p* < 0.05 or *p* < 0.01) expression levels of osteogenic genes than those in the control and CGRP-BIBN group after incubation for 14 days, including ALP, Runx2, Col I, OPN, OCN, and OPG.

### 3.5. Proosteogenic Effect by cAMP/PKA Signaling Pathway

**Figure 3:**
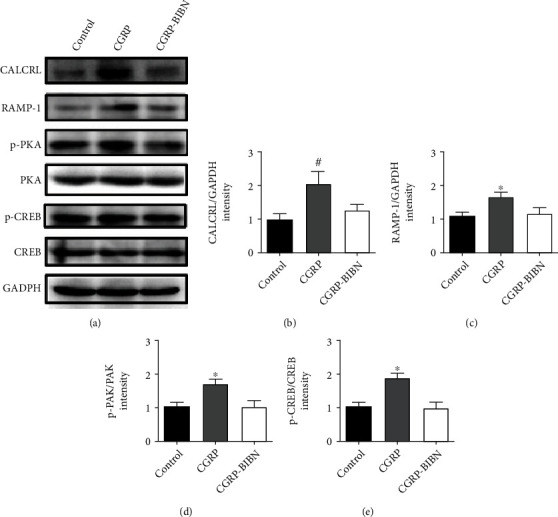
Western blotting of CGRP-related proteins. (a) Western blotting and (b)–(e) quantitative analysis indicated that CGRP promoted the protein contents of CALCRL, RAMP-1, pPKA/PKA, and pCREB/CREB. ^∗^*p* < 0.05, ^#^*p* < 0.01, as compared with control and CGRP-BIBN groups (*n* = 6).

To further explore the potential underlying molecular mechanism of CGRP for its proosteogenic capacity, western blotting was performed to measure the expression levels of certain proteins. For western blotting assay (Figure 3**)**, we observed that the CALCRL, RAMP-1, and phosphorylation levels of PKA and CREB in MSCs treated with CGRP were significantly higher (*p* < 0.05 or *p* < 0.01) than that of control group. However, the promotion effects were significantly inhibited via adding CGRP-receptor antagonist BIBN4096BS in the CGRP-BIBN group.

### 3.6. CGRP Promotes Accumulations of F-Actin Filaments

**Figure 4:**
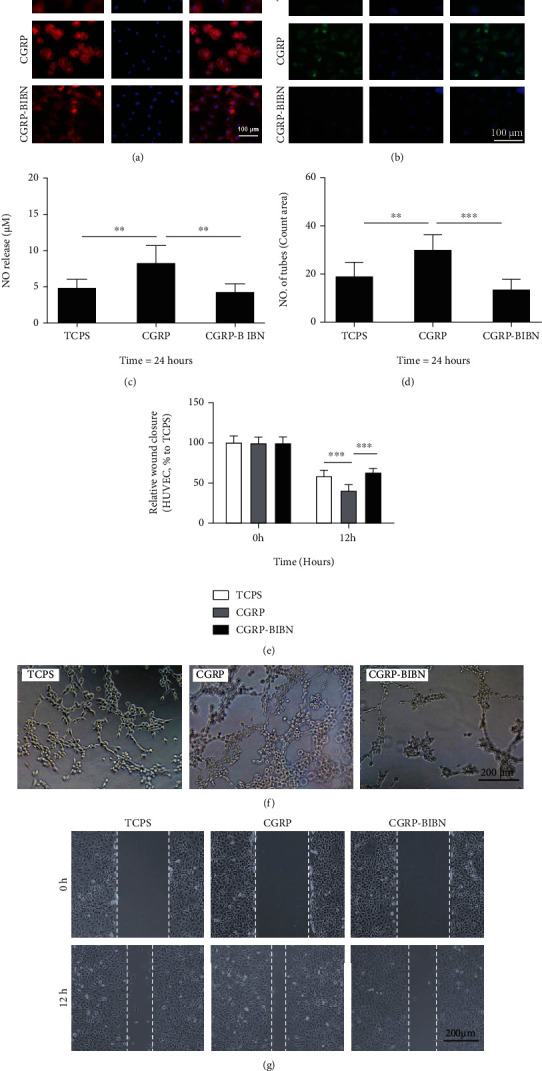
CGRP enhances angiogenesis of HUVECs. (a) F-actin staining and (b) iNOS staining of HUVECs; (c) NO release of HUVEC (*n* = 12); (d) quantitative measurement and (f) representative images of tube formation by HUVECs after 24-hour incubation (*n* = 6); (e) quantitative evaluation and representative images wound healing closure of HUVECs (*n* = 6); ∗*p* < 0.05, ∗∗*p* < 0.01, and ^∗∗∗^*p* < 0.001, as compared with control and CGRP-BIBN groups.

To evaluate the proangiogenic features of CGRP, the morphology of HUVECs was firstly detected via F-actin cytoskeleton staining (Figure 4(a)). After 24 hours of incubation, CGRP was observed to promote accumulations of F-actin filaments in HUVECs in the CGRP group as compared with the TCPS group. However, the administration of BIBN4096BS resulted in the loss of actin stress fibers in HUVECs in the CGRP-BIBN group.

### 3.7. CGRP Promotes iNOS Activity of HUVECs

iNOS is well known to regulate angiogenesis of endothelial cells (EC) via promoting excess nitric oxide (NO) production, a gaseous molecule that plays many key roles in the maintenance of endothelial cell growth and angiogenesis. To evaluate the regulation effect of CGRP on iNOS activity in HUVECs, the iNOS expression was firstly detected with immunofluorescence staining. As shown in Figure 4(b), compared with the TCPS group, HUVECs in the CGRP group were observed with high levels of iNOS expression, which could be reduced via BIBN4096BS administration in the CGRP-BIBN group. NO release results confirmed that higher level of iNOS expression in the CGRP group resulted in higher level of NO release as compared with the TCPS group, and the increasing regulation effect of CGRP on NO release was inhibited via BIBN4096BS administration in the CGRP-BIBN group (*p* < 0.05 or *p* < 0.01).

### 3.8. CGRP Ameliorated Tube Formation and Wound Healing of HUVECs

To explore the effect of CGRP on endothelial migration and tube formation, tube formation and wound healing assay of HUVECs were performed. As shown in Figures 4(d) and 4(f), incubation with CGRP stimulated increased vessel formation numbers in CGRP group as compared to TCPS group (*p* < 0.05 or *p* < 0.01). Conversely, the promotion effect was inhibited in BIBN4096BS administrated the CGRP-BIBN group. Similarly, compared with the TCPS group, the scratch wound healing process results showed that CGRP could significantly accelerate wound healing of HUVECs in the CGRP group (*p* < 0.05 or *p* < 0.01), and the promotion effect of CGRP was inhibited via BIBN4096BS (Figures 4(e) and 4(g)). These results indicated that CGRP has the potential to induce angiogenesis of HUVECs.

### 3.9. CGRP Enhances Proangiogenic Features of MSCs

**Figure 5:**
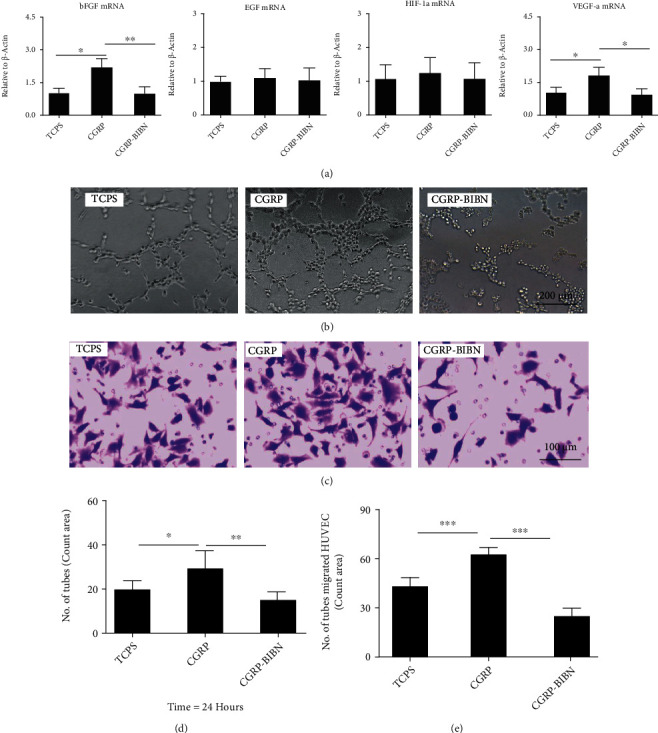
CGRP enhances proangiogenic features of MSCs and indirectly contributes to increased angiogenesis effects of HUVECs. (a) RT-qPCR measurement of proangiogenic gene expression levels in MSCs (*n* = 6). (b) Representative images of tube formation by HUVECs cultured in MSC-derived conditioned medium (CM) and (d) its quantitative measurement accordingly (*n* = 6). (c) Representative images of transmembrane migrated HUVECs cocultured with MSCs and (e) its quantitative measurement accordingly (*n* = 6); ^∗^*p* < 0.05, ^∗∗^*p* < 0.01, ^∗∗∗^*p* < 0.001, as compared with control and CGRP-BIBN groups.

In order to identify potential MSC-derived signals that may act on HUVECs, proangiogenic gene expression levels of MSCs were firstly detected by RT-qPCR. The results showed that CGRP dramatically enhanced the mRNA expression of bFGF and VEGF-a in MSCs at 3 days after incubation, and the increased gene expression was inhibited in the CGRP-BIBN group (Figure 5(a), *p* < 0.05 or *p* < 0.01).

To further investigate the paracrine effects of MSCs on tube formation of HUVECs, we firstly treated MSC with different conditions to obtain the MSC-derived conditioned medium (CM). The CM from different groups was applied for tube formation assay of HUVECs. As shown in Figures 5(b) and 5(d), HUVEC incubated with CM from CGRP group was observed with larger number of tube formation compared with TCPS and CGRP groups (*p* < 0.05 or *p* < 0.01). Transwell-based coculture experiment result showed that MSC from the CGRP group could dramatically enhance transmembrane migration of HUVECs when compared with the TCPS group, and the transmembrane migration effect could be inhibited in the CGRP-BIBN group (Figures 5(c) and 5(e), *p* < 0.05 or *p* < 0.01). These results further confirmed that CGRP could enhance proangiogenic features of MSCs, which might contribute to enhanced angiogenesis of HUVECs.

## 4. Discussion

In the bone tissue, sensory nerves have been highlighted for their potential to heal damaged bone tissue via some released neuropeptides. Some investigations about bone regeneration, or even bone tissue engineering, have pointed out the potential of calcitonin gene-related polypeptide-*α* (CGRP) in promoting osteogenic differentiation effects of bone metabolism-related cells *in vitro* [11] and bone formation *in vivo* [25, 26]. However, the related mechanisms of bone regeneration regulation effects of CGRP are still not well clarified so far, in particular, for its potential roles in the process of coupled osteogenesis and angiogenesis regulation during bone healing.

In this study, we firstly provided some previously unrecognized results for an optimum concentration and the ability of enhanced MSCs proliferation and osteogenic differentiation regulation effect of CGRP. Our studies supported the point of view that high concentration of CGRP might inhibit the proliferation of MSCs **(**Figure 1(a)**)**. No decrease in cell viability of MSCs by the higher concentration of CGRP (up to 10^−8^ M) was observed, and the inhibitory effect was more obvious when the concentration rise to 10^−7^ M. The optimum concentration of CGRP for proliferation was similar to a previous work in periosteum-derived stem cells (PDSCs) [12].

Previously papers indicated that CGRP could trigger various intracellular signaling cascades involving cyclic adenosine monophosphate (cAMP) via binding to G protein-coupled receptor composited by calcitonin receptor-like receptor (CRLR) and receptor activity modifying protein 1 (RAMP1) on cellular surface [11]. According to the report by Vignery and McCarthy, CGRP could induce the cAMP accumulation in osteoblastic cells [27]. By contrast, Drissi et al. reported the absence of cAMP accumulation in osteosarcoma cells (OHS-4) after CGRP treatment [28]. Therefore, the levels of cAMP in MSCs incubated with different concentrations of CGRP were assayed first in this study. Our results showed that cAMP level was significantly promoted when MSCs were treated with the high concentration of CGRP (10^−10^–10^−7^ M); however, the most appropriate concentration of CGRP is 10^−9^ M (Figure 1(b)).

ALP activity and mineralization were employed to reflect the differentiation of osteoblasts at different periods. In this study, in order to study the role of CGRP in osteogenic differentiation of MSCs, the BIBN4096BS, a CGRP-receptor antagonist, was used to screen the effects of CGRP. As shown by ALP and ARS staining, the CGRP promoted the ALP activity and mineralization. However, CGRP-receptor antagonist cotreatment could effectively inhibit the differentiation promotion of CGRP. The runt-related transcription factor 2 (RUNX2) is an essential transcription factor of early osteoblast differentiation and the master gene of bone regeneration [29]. Our data showed that activation of the CGRP receptor could initiate the cAMP-signaling pathway, which leads to the subsequently activation of RUNX2. Activation of RUNX2 could lead to a further enhanced expression of downstream osteogenic genes (Col I, OPN, OCN, ALP, etc.). In this study, qRT-PCR results showed that CGRP (10^−9^ M) treatment resulted in statistically upregulated of all these osteogenic related genes at molecular level. Western blotting results confirmed that CGRP could enhance the expression of CALCRL, RAMP-1, and the phosphorylation levels of PKA and CREB in MSCs, which further indicated the potential regulatory role of cAMP/PKA signaling pathway in the proosteogenic effect of CGRP. Taken together, these results revealed the proosteogenic effects of CGRP and the underlying molecular mechanism.

Large numbers of studies have highlighted the vital roles of coupled angiogenesis and osteogenesis during bone healing process, particular for the cross-talk between endothelial cells and osteogenic cells [30, 31]. Recently, papers also demonstrated that, apart from proosteogenesis, CGRP also displayed a potential promotive role in angiogenesis and vasodilatory of injury bone site, which in turn indirectly promote osteogenesis [13, 32]. In this study, our results proved that CGRP could directly increase endothelial NO synthase activity, wound healing ability, and functional capillary formation of HUVECs, which contribute to enhanced angiogenesis. Besides, CGRP was also proved to be able to enhance proangiogenic features of MSCs via increasing bFGF and VEGF-a proangiogenic gene expressions, which indirectly enhance tube formation and transmembrane migration of HUVECs via MSCs' paracrine effects.

Despite these results, in this study, we firstly used MSC cell viability detection and the levels of cAMP to evaluate the concentration-based regulation role of CGRP in MSCs and screen appropriate treatment concentration for the following experiments. Based on MSC cell viability detection and cAMP results, 10-9 M is the critical value of CGRP and thus be used for the following study. Even so, it cannot rule out that CGRP might have a special concentration-based regulation role of CGRP in HUVECs. Accordingly, our ongoing studies will address more on the potential regulation effects of CGRP in HUVECs during osteogenesis or bone healing process.

## 5. Conclusion

**Scheme 1:**
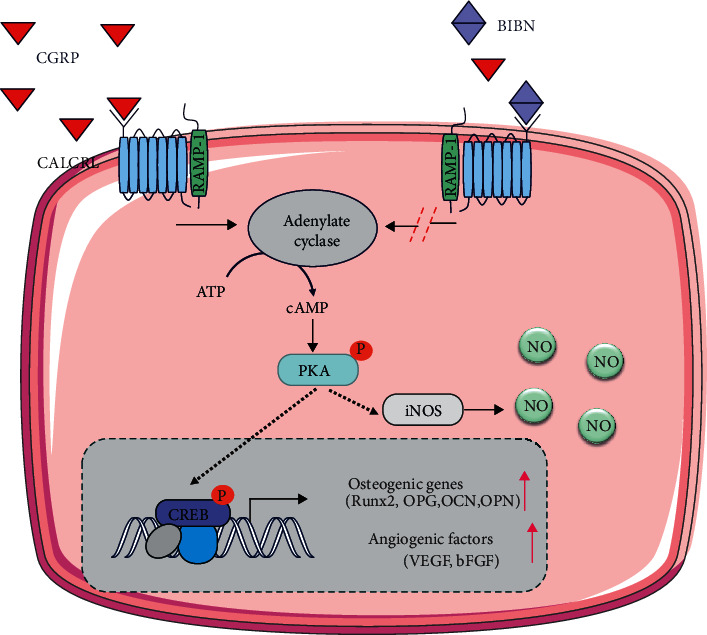
Molecular mechanisms mediating coregulated angiogenesis and osteogenesis effects of CGRP.

In conclusion, CGRP-induced osteogenic differentiation of MSCs has been investigated and shown effective potential. In particular, it could induce the cAMP accumulation and further promote the ALP activity and mineralization of MSCs. In addition, activation of the cAMP/PKA signaling pathway leads to activate RUNX2, which subsequently regulates the transcription of downstream osteogenic genes. What is more important, CGRP could also enhance angiogenesis directly via endothelial cells or indirectly via enhancing proangiogenic features of MSCs Scheme 1. Therefore, the present findings prove the feasibility of using CGRP as functional drug to coregulate the osteogenic and angiogenic response, which could improve bone tissue regeneration in clinical application.

## Figures and Tables

**Table 1:** Real-time PCR primers used in this study.

Target gene	Genebank (accession no.)	Primers	Product size (bp)
*β*-Actin	NM_031144.2	GGAGATTACTGCCCTOGCTCCTA GACTCATCGTACTCCTGCTTGCTG	150
Runx2	NM_053470.2	GCCGTAGAGAGCAGGGAAGAC CTGGCTTGGATTAGGGAGTCAC	150
ALP	NM_013059	AGCGACACGGACAAGAAGC GGCAAAGACCGCCACATC	183
Col I	NM_053304.1	CCTGAGCCAGCAGATTGA TCCGCTCTTCCAGTCAG	106
OCN	M11777	GAGGGCAGTAAGGTGGTGAA CGTCCTGGAAGCCAATGTG	154
OPN	M99252	GACAGCAACGGGAAGACC CAGGCTGGCTTTGGAACT	216
OPG	RUN94330	GCCCAGACGAGATTGAGAG CAGACTGTGGGTGACGGTT	173
bFGF	NM_001361665.2	TCCACCTATAATTGGTCAAAGTGGT CATCAGTTACCAGCTCCCCC	121
EGF	NM_001963.6	TGGTGATGGGAGGATGACTTG GGCCAGTGACTCAGCAGAAA	158
HIF-1a	AB733094.1	AGCTTGCTCATCAGTTGCCA CCAGTTAGTTCAAACAGCATCCA	125
VEGF-a	BC065522.1	CCCACTGAGGAGTCCAACATC CGGCTTGTCACATTTTTCTTGTC	150

## Data Availability

The statistical data of the article used to support the findings of this study are available from the corresponding author upon request.
